# Gestational weight gain and adverse birth outcomes in South African women with HIV on antiretroviral therapy and without HIV: a prospective cohort study

**DOI:** 10.1002/jia2.26313

**Published:** 2024-06-26

**Authors:** Hlengiwe P. Madlala, Landon Myer, Jennifer Jao, Hayli Geffen, Mushi Matjila, Azetta Fisher, Demi Meyer, Erika F. Werner, Gregory Petro, Susan Cu‐Uvin, Stephen T. McGarvey, Angela M. Bengtson

**Affiliations:** ^1^ Division of Epidemiology and Biostatistics School of Public Health, University of Cape Town Cape Town South Africa; ^2^ Division of Infectious Diseases, Department of Pediatrics Northwestern University Feinberg School of Medicine Chicago Illinois USA; ^3^ Division of Infectious Diseases, Department of Medicine Northwestern University Feinberg School of Medicine Chicago Illinois USA; ^4^ Department of Obstetrics & Gynaecology University of Cape Town, Groote Schuur and New Somerset Hospitals Cape Town South Africa; ^5^ Department of Obstetrics and Gynaecology School of Medicine, Tufts University Boston Massachusetts USA; ^6^ Department of Obstetrics and Gynaecology and Medicine Warren Alpert School of Medicine, Brown University Providence Rhode Island USA; ^7^ Department of Epidemiology and International Health Institute School of Public Health, Brown University Providence Rhode Island USA; ^8^ Department of Epidemiology Rollins School of Public Health, Emory University Atlanta Georgia USA

**Keywords:** gestational weight gain, HIV, dolutegravir, birthweight, size for gestational age, antiretroviral therapy

## Abstract

**Introduction:**

Outside of pregnancy, evidence shows that persons with HIV initiating or switching to dolutegravir (DTG)‐based antiretroviral therapy (ART) experience greater weight gain compared to those on other ART classes. However, there are few data on the impact of DTG‐based ART on gestational weight gain (GWG) in sub‐Saharan Africa where HIV is most common. According to the National Academy of Medicine (NAM), GWG below and above NAM guidelines is associated with adverse birth outcomes. Therefore, the objective of this study was to describe GWG by HIV status and ART regimen, and examine the associations with adverse birth outcomes.

**Methods:**

We enrolled pregnant women with HIV (WHIV) and without HIV (≥18 years) in a peri‐urban primary healthcare facility in Cape Town, South Africa between 2019 and 2022. GWG was study‐measured at 24–28 (baseline) and 33–38 weeks gestation and converted to GWG rate (kg/week) in accordance with NAM guidelines. GWG z‐scores were generated using the INTEGROWTH‐21 and US standards to account for differing lengths of gestation. Birth outcome data were obtained from medical records. Associations of GWG z‐score with adverse birth outcomes were assessed using multivariable linear or log‐binomial regression.

**Results:**

Among 292 participants (48% WHIV), median age was 29 years (IQR, 25–33), median pre‐pregnancy body mass index (BMI) was 31 kg/m^2^ (IQR, 26–36) and 20% were primiparous at baseline. The median weekly rate of GWG was 0.30 kg/week (IQR, 0.12–0.50), 35% had GWG below NAM standards (59% WHIV) and 48% had GWG above NAM standards (36% WHIV). WHIV gained weight more slowly (0.25 vs. 0.37 kg/week, *p*<0.01) than women without HIV. Weekly rate of GWG did not differ by ART regimen (DTG‐based ART 0.25 vs. efavirenz‐based ART 0.27 kg/week, *p* = 0.80). In multivariable analyses, GWG z‐score was positively associated with continuous birth weight (mean difference = 68.53 95% CI 8.96, 128.10) and categorical high birth weight of >4000 g (RR = 2.18 95% CI 1.18, 4.01).

**Conclusions:**

Despite slower GWG among WHIV, nearly half of all women gained weight faster than recommended by the NAM. GWG was positively associated with infant birth weight. Interventions to support healthy GWG in sub‐Saharan Africa are urgently needed.

## 1 INTRODUCTION

In line with the World Health Organisation recommendations, in 2019 South Africa implemented dolutegravir (DTG)‐based antiretroviral therapy (ART) as the preferred first‐line ART regimen for all persons with HIV (PHIV), including pregnant women [1, 2]. Outside of pregnancy, evidence shows that PHIV initiating DTG‐based ART experience greater weight gain compared to those initiating other ART classes [3, 4]. In particular, participants in randomized controlled trials in South Africa and Cameroon gained significantly more weight at 1 and 2 years post DTG‐based ART initiation versus those initiating efavirenz (EFV)‐based ART, with the largest gains seen among women [5, 6]. In addition, the African cohort study reported higher weight change in participants switching to DTG‐based ART compared to other ART regimens [7]. In pregnancy, the Tsepamo study in Botswana found that PHIV on DTG‐based ART had higher weekly gestational weight gain (GWG) than those on EFV‐based ART [8]. However, there are limited data in other sub‐Saharan African countries.

Outside the context of ART regimen, WHIV have been shown to experience inadequate GWG, partly attributed to HIV disease progression, lower socio‐economic status (SES) and higher levels of food insecurity compared to women without HIV [9, 10]. Although GWG is a normal physiological process in pregnancy, gaining too much or too little can adversely affect maternal and infant health outcomes. As a result, the National Academy of Medicine (NAM) (formerly known as the Institute of Medicine) developed GWG guidelines to assist clinical practice with the identification of women at risk of adverse outcomes [11]. GWG below NAM guidelines is linked to an increased risk of preterm delivery (PTD) and small size for gestational age (SGA) infants [11]. In contrast, GWG above recommendations increases the risk of high birth weight (HBW) and large size for gestational age (LGA) in infants [12, 13, 14]. In addition, GWG above the NAM guidelines is associated with gestational diabetes, hypertension, pre‐eclampsia and a long‐term risk of obesity and cardiovascular diseases (CVDs) in women [15, 16, 17]. Recently, the Collaborative Perinatal Project Mortality Linkage Study of 46,042 participants followed for over 50 years showed that GWG above NAM recommendations was associated with increased all‐cause, diabetes‐ and CVD‐related mortality [18]. Consequently, there are increasing concerns regarding the implication of the collision of HIV and DTG‐associated weight gain on cardiometabolic health outcomes of pregnant women and their children.

Despite the high burden of HIV in sub‐Saharan Africa, there are limited data on DTG‐based ART and GWG, and subsequent impacts on birth outcomes. To address this gap, we conducted a prospective cohort study in pregnant women with HIV (WHIV, using DTG‐ or EFV‐based ART) and without HIV attending an antenatal care clinic in Cape Town. The primary objective of this study was to describe GWG by HIV status and ART regimen, and examine the associations with subsequent adverse birth outcomes.

## 2 METHODS

### 2.1 Study design and population

A total of 400 pregnant women (≥18 years of age, 24–28 week's gestation) were enrolled from a maternity obstetric unit (MOU) at Gugulethu primary healthcare facility. Gugulethu is a township situated 18 km south‐east of Cape Town in the Western Cape Province, South Africa. This township has a population of approximately 300,000 that is predominantly made up of Black African ethnic group (98.8%), with low SES receiving an average monthly income of ≤ $166 USD [19, 20]. The MOU provides antenatal and obstetric care for low‐risk pregnancies without complications to approximately 5000 women per annum. HIV care is integrated into antenatal and obstetric care, including the provision of ART at no cost. Current South African maternity guidelines do not include a recommendation for monitoring GWG for pregnant women [21].

Study recruitment took place between November 2019 and June 2022. This time period coincided with the switching of first‐line ART from EFV‐based (tenofovir 300 mg + [emtricitabine 200 mg or lamivudine 300 mg] + EFV 600 mg) to DTG‐based (tenofovir 300 mg + [emtricitabine 200 mg or lamivudine 300 mg] + DTG 50 mg). As a result, WHIV were either on EFV‐based ART (pre‐conception or post‐conception) or DTG‐based ART (mostly post‐conception). This distinction of the timing of ART initiation was important for this study in order to understand how the timing of ART initiation in pregnancy could affect GWG.

**Figure 1:**
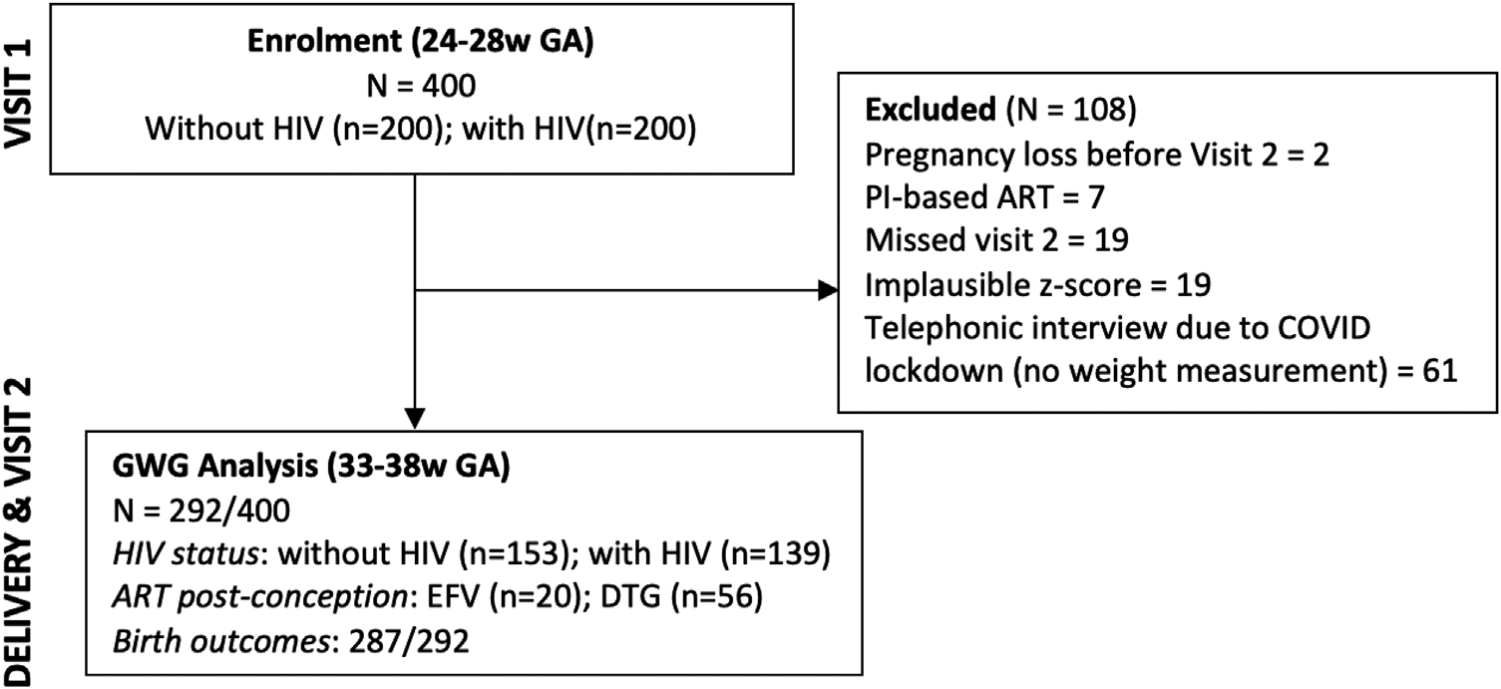
Participant enrolment and retention flowchart showing the selection of participants included in the analysis. ART, antiretroviral therapy; BMI, body mass index; DTG, dolutegravir; EFV, efavirenz; PI, protease inhibitor.

Participants were followed from 24–28 weeks gestation (baseline) to 33–38 weeks gestation, delivery (medical record abstraction) and 6 months postpartum through face‐to‐face study visits. We conducted a GWG analysis which included all participants with valid weight measurements at both 24–28 and 33–38 weeks gestational age (GA) (*n* = 292) and complete birth outcome data (*n* = 287) (Figure 1). We compared rates of GWG by HIV status and post‐conception ART regimen. A comparison of GWG among pre‐conception ART initiators was not conducted because of the few women (*n* = 9) who were on DTG prior to conception due to the recency of the rollout during study recruitment. The study protocol was reviewed and approved by the Faculty of Health Sciences Human Research Ethics Committee of the University of Cape Town (FHS‐HREC 486/2019 and 505/2020) and the Western Cape Department of Health (WC_202011_024). All women provided written informed consent prior to study participation.

## 3 MEASURES

### 3.1 Exposure assessment

The exposures were HIV status, ART regimen and GWG. HIV status and ART regimen were collected through self‐report and confirmed in the medical records. Weight was measured at 24–28 and 33–38 weeks gestation by a trained study nurse using a calibrated scale (Charder, Taichung City, Taiwan) accurate to within 0.2 kg. GWG was calculated as the average weekly rate of GWG between the second and third trimesters in accordance with NAM guidelines: (33–38 weeks gestation weight)—(24–28 weeks gestation weight) / (weeks between 24–28 and 33–38 weeks gestation). We only assessed the weekly rate of GWG at the 24–28 and 33–38 week time points because the NAM guidelines are only defined for the second and third trimesters since that is when most GWG occurs [11]. Weekly rate of GWG was then categorized as below, within or above NAM standards, based on women's pre‐pregnancy body mass index (BMI) [11].

We used GWG rate to describe differences in weekly rate of GWG by HIV status and post‐conception ART regimen. In sensitivity analysis, we assessed weekly rate of GWG among all ART users. For the associations between GWG and adverse birth outcomes, we used GWG z‐scores which standardize weight assessments taken at different GAs and, therefore, is less biased for outcomes defined by GA (e.g. preterm birth, small‐for GA) [22]. Global standards of GWG are not available for women entering pregnancy with overweight or obesity, or for WHIV. Therefore, we generated the GWG z‐scores using the INTERGROWTH‐21 standards for normal pre‐pregnancy BMI and US standards for overweight and obese pre‐pregnancy BMI [23, 24]. GWG z‐scores were divided into tertiles and categorized as lower, medium and higher GWG. For comparability, we assessed the association between weekly rate of GWG and adverse birth outcomes in sensitivity analysis.

### 3.2 Outcome assessment

The birth outcomes included PTD, birthweight (continuous, low birth weight and high birth weight) and size for GA (small and large for GA). GA at enrolment was primarily determined by an ultrasound (83%) operated by an experienced research sonographer, the remaining 17% were assessed by either symphysis fundal height measurement or last menstrual period. GA at delivery was categorized as term (≥37 weeks) or preterm (PTD, <37 weeks) [25]. Infant birth weight was categorized as low (<2500), normal (2500–4000) and high (>4000), in grams [26]. Birth weight for GA was calculated using INTERGROWTH‐21st standards [27], and categorized as small for GA (SGA, <10th percentile), appropriate for GA (AGA, 10–90th percentile) or large for GA (LGA, >90th percentile).

### 3.3 Covariates

Maternal socio‐demographic and clinical data were collected via trained interviewer‐administered questionnaires at baseline. SES was a composite score based on level of education, employment status, type of housing and presence of a toilet, running water, electricity, fridge, telephone, and television in the house [28]; participants were categorized into tertiles corresponding to lower, middle and higher SES group. Alcohol use was measured using a 3‐item Alcohol Use Disorders Identification Test‐Consumption (AUDIT‐C; range 0–12). An AUDIT‐C score ≥3 indicates hazardous drinking in the previous 12 months for women [29]. Perceived household food insecurity was assessed using a measure adapted from the Household Food Insecurity Access Scale and was categorized as “yes” and “no” [30]. Among WHIV, CD4 count and viral load measurements within 6 months before enrolment in the study were abstracted from medical records. Pre‐pregnancy weight was self‐reported and used to calculate pre‐pregnancy BMI as weight divided by squared height, categorized as underweight (<18.5), normal (18.5–24.9), overweight (25.0–29.9) and obese (≥30.0) in kg/m^2^. Although other studies suggest underreporting of pre‐pregnancy weight, particularly among individuals with overweight and obesity [31], others have shown that pre‐pregnancy BMI categories from self‐reported versus measured pre‐pregnancy weights are similar [32].

### 3.4 Statistical analysis

Differences in weekly rate of GWG by HIV status and post‐conception ART regimen were visualized graphically. Log‐binomial models were fit to estimate the associations of GWG z‐scores (primary analysis) and GWG rate (sensitivity analysis), with adverse birth outcomes. Linear regression was used to evaluate associations between the same set of exposures and continuous birth weight. Potential confounders (age, SES, food insecurity, CD4 count) included in the multivariable models were based on literature [9, 10], theoretical or conceptual reasoning. In addition, we graphically explored differences in GWG z‐score tertiles and adverse birth outcomes by HIV status and ART regimen, since limited sample size precluded statistical assessment of effect measure modification. All data were analysed using STATA version 15.0 (Stata Corporation, College Station, TX, USA) and R Studio (R Foundation, Vienna, Austria).

## 4 RESULTS

A total of 292 women (48% WHIV) were assessed for GWG in pregnancy. At study enrolment, the median age was 29 years (IQR, 25–33), GA was 26 weeks (IQR, 24–27) and 20% of women were primiparous (Table 1). WHIV were older (31 vs. 27 years, *p*<0.01), had lower tertiary education (4 vs. 7%, *p* = 0.03) and were less likely to be primiparous (12 vs. 27%, *p*<0.01) compared to those without HIV. Among WHIV, 26% (*n* = 76) initiated ART post‐conception (DTG 74% [*n* = 56]; EFV 26% [*n* = 20]). The median pre‐pregnancy BMI was 31 kg/m^2^ (IQR, 26–36): 0 (0%) underweight, 45 (15%) normal, 82 (28%) with overweight and 165 (57%) with obesity. Of the 165 women with obesity, 90 (55%) did not have HIV and 75 (45%) were WHIV. Women included in the overall cohort (*n* = 400) had similar demographic characteristics as the subset cohort for GWG analysis (Table S1).

**Table 1:** Characteristics of women included in the GWG analysis, overall and stratified by maternal HIV status

		HIV status		
Baseline characteristic	Subset with GWG *N* = 292	Without HIV *N* = 153	With HIV *N* = 139	*p*‐value
Age (years)				
Median (IQR)	29 (25–33)	27 (24–31)	31 (27–36)	<0.01
Gestational age at first ANC (weeks)				
Median (IQR)	17 (12–21)	17 (13–21)	17 (12–21)	0.36
Gestational age at study enrolment (weeks)				
Median (IQR)	26 (24–27)	26 (24–27)	26 (24–27)	0.80
Weight (kg), median (IQR)				
Pre‐pregnancy	80 (65–93)	80 (68–90)	78 (65–95)	0.39
24–28 weeks GA	84 (71–98)	86 (74–98)	81 (69–98)	0.21
33–38 weeks GA	87 (75–100)	89 (78–101)	83 (71–100)	0.04
Pre‐pregnancy BMI (kg/m^2^)				0.30
Underweight (<18.5)	0 (0)	0 (0)	0 (0)	
Normal (18.5–24.9)	45 (15)	23 (15)	22 (16)	
Overweight (25–29.9)	82 (28)	40 (26)	42 (30)	
Obese (≥30)	165 (57)	90 (59)	75 (54)	
Median (IQR)	31 (26–36)	31 (27–36)	31 (26–36)	
Parity				<0.01
Primiparous	58 (20)	42 (27)	16 (12)	
Multiparous	234 (80)	111 (73)	123 (88)	
Median (IQR)	2 (2–3)	2 (1–3)	3 (2–4)	
Education				0.03
Primary	5 (1)	2 (1)	3 (2)	
Partially completed high school	177 (61)	81 (53)	96 (69)	
Completed high school	93 (32)	59 (39)	34 (25)	
Tertiary	17 (6)	11 (7)	6 (4)	
Socio‐economic status				0.13
Lower	96 (33)	44 (29)	52 (37)	
Middle	76 (26)	38 (25)	38 (28)	
Higher	120 (41)	71 (46)	49 (35)	
Relationship status				0.10
Not in relationship	24 (8)	8 (5)	16 (11)	
Not married/cohabiting	143 (49)	81 (53)	62 (45)	
Married/cohabiting	125 (43)	64 (42)	61 (44)	
Perceived food insecurity				0.15
No	241 (83)	131 (86)	110 (79)	
Yes	51 (17)	22 (14)	29 (21)	
Hazardous alcohol use				0.78
No	274 (94)	143 (93)	131 (94)	
Yes	18 (6)	10 (7)	8 (6)	
^a^Gestational weight gain (kg/week)				<0.01
Below NAM standards	101 (35)	41 (27)	60 (43)	
Within NAM standards	50 (17)	22 (14)	28 (20)	
Above NAM standards	141 (48)	90 (59)	51 (37)	
Median (IQR)	0.30 (0.12–0.50)	0.37 (0.22–0.63)	0.25 (0.08–0.42)	

Maternal HIV characteristics				
	Subset with GWG *N* = 13	EFV‐based ART (*n* = 75)	DTG‐based ART (*n* = 64)	*p*‐value
ART initiation timing				<0.01
Pre‐conception	63 (45)	55 (73)	8 (12)	
Post‐conception	76 (55)	20 (27)	56 (88)	
Duration on ART (months)				<0.01
Median (IQR)	3 (1–50)	46 (4–84)	1 (1–2)	
CD4 count (cells/µl)				<0.01
≤350	27 (19)	15 (20)	12 19)	
351–500	30 (22)	20 (27)	10 (16)	
>500	52 (37)	33 (44)	19 (30)	
Median (IQR)	463 (352–692)	478 (358–695)	461 (260–630)	
Viral load (copies/ml)				<0.01
Undetectable (<50)	45 (32)	44 (59)	1 (2)	
Detectable (≥50)	19 (14)	13 (17)	6 (9)	
Median (IQR)	15 (15–97)	15 (15–20)	3380 (212–253,301)	
Weight (kg), median (IQR)				
Pre‐pregnancy	78 (65–95)	78 (65–95)	78 (66–94)	0.87
24–28 weeks GA	81 (69–98)	83 (71–99)	79 (69–98)	0.42
33–38 weeks GA	83 (71–100)	86 (72–100)	82 (70–98)	0.38
Gestational weight gain (kg/week)				0.67
Below NAM standards	60 (43)	34 (46)	26 (41)	
Within NAM standards	28 (20)	16 (21)	12 (18)	
Above NAM standards	51 (37)	25 (33)	26 (41)	
Median (IQR)	0.25 (0.08–0.43)	0.26 (0.03–0.42)	0.25 (0.11–0.42)	

*Note*: Missing data on overall sample *n* = 400: Missing data: gestational age at first ANC *n* = 8, pre‐pregnancy weight *n* = 1, pre‐pregnancy BMI *n* = 1, gestational weight gain *n* = 108, CD4 count *n* = 41, viral load *n* = 111. Missing data on subset *n* = 292: gestational age at first ANC *n* = 6, CD4 count *n* = 30, viral load *n* = 75.

Abbreviations: ANC, antenatal care; ART, antiretroviral therapy; BMI, body mass index; DTG, dolutegravir; EFV, efavirenz; GA, gestational age; NAM, National Academy of Medicine.

### 4.1 GWG analysis

**Figure 2:**
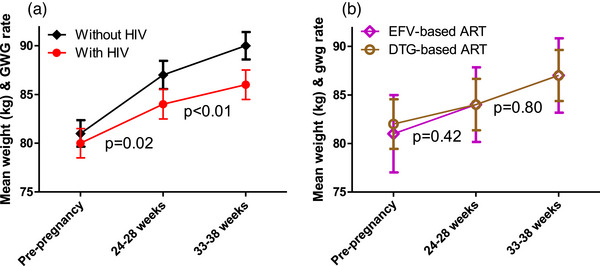
Weekly rate of GWG by HIV status (A: 153 without HIV, 139 WHIV) and post‐conception ART (B: 20 EFV‐based ART, 56 DTG‐based ART). The three time points on the plot show mean weights at pre‐pregnancy (self‐reported), 24–28 weeks gestation (measured) and 33–38 weeks gestation (measured), and the lines connecting the dots represent weekly rate of GWG. *p*‐value < 0.05 represents significant difference in weekly rate of GWG slopes of the comparison groups. ART, antiretroviral therapy; DTG, dolutegravir; EFV, efavirenz.

Among women with complete data on GWG (*n* = 292), overall mean weight at 24–28 weeks gestation was 85 kg (SD = 18) and 88 kg (SD = 18) at 33–38 weeks gestation. There was no difference in weight at 24–28 weeks gestation by HIV status (WHIV 84 vs. without HIV 87 kg, *p* = 0.21), but WHIV weighed less at 33–38 weeks gestation compared to women without HIV (WHIV 86 vs. without HIV 90 kg, *p* = 0.04) (Figure 2A). The median weekly rate of GWG was 0.30 kg/week (IQR, 0.12–0.50): 101 (35%) had GWG below, 50 (17%) within and 141 (48%) above NAM recommended weekly rate of GWG for their pre‐pregnancy BMI. Of the 101 women with weekly rate of GWG below NAM recommendations, 41 (41%) did not have HIV and 60 (59%) had HIV. Of the 141 women with weekly rate of GWG above NAM recommendations, 90 (64%) did not have HIV and 51 (36%) had HIV. WHIV gained weight slower between 24–28 weeks gestation and 33–38 weeks gestation than women without HIV (WHIV 0.25 vs. without HIV 0.37 kg/week, *p*<0.01).

Among post‐conception ART initiators, women on DTG‐based ART had similar mean weight at 24–28 weeks gestation (DTG‐based ART 84 kg SD = 17 vs. EFV‐based ART 84 kg SD = 20, *p* = 0.89) and 33–38 weeks gestation (DTG‐based ART 87 kg SD = 17 vs. EFV‐based ART 87 kg SD = 20, *p* = 0.76) as those on EFV‐based ART (Figure 2B). Post‐conception DTG‐based ART initiation did not lead to a higher weekly rate of GWG between 24–28 weeks gestation and 33–38 weeks gestation compared to EFV‐based ART (DTG‐based ART 0.24 vs. EFV‐based ART 0.30 kg/week, *p* = 0.80). In sensitivity analysis, weekly rate of GWG was also similar among all ART users (both pre‐ and post‐conception; DTG‐based ART 0.26 vs. EFV‐based ART 0.28 kg/week, *p* = 0.84).

The distribution of GWG z‐scores were in the same direction as GWG rate (Figures S1 and S2). When GWG was converted to z‐scores and categorized as tertiles, women in the lower z‐score tertile had a median weekly rate of GWG of 0.25 kg/week (IQR 0.06, 0.34), those in the medium z‐score tertile had a median weekly rate of GWG of 0.29 kg/week (IQR 0.11, 0.48) and those in the higher z‐score tertile had a median weekly rate of GWG of 0.44 kg/week (IQR 0.25, 0.76) (Figure S3). There were no differences in GWG z‐score tertile and median weekly rate of GWG relationship by HIV status.

### 4.2 Birth outcome analysis

Overall, 13% of infants had LBW, 4% HBW, 8% SGA, 15% LGA and 17% were preterm.

In multivariable analyses, a one‐unit increase in GWG z‐score was associated with a mean increase of 68.53 grams in birth weight (95% CI 8.96, 128.10), *p* = 0.02. A one‐unit increase in GWG z‐score was also associated with an increased risk of HBW (aRR = 2.18 95% CI 1.18, 4.01), *p* = 0.01. When GWG z‐scores were categorized into tertiles, higher GWG z‐score tertile was associated with increased birth weight (mean difference [MD] = 158.15 95% CI 20.24, 296.07), *p* = 0.02 compared to medium tertile (Table 2). Higher GWG z‐score tertile was also associated with increased LGA (aRR = 1.22 95% CI 0.65, 2.29) and decreased SGA (aRR = 0.62 95% CI 0.21, 1.82), though these associations did not reach significance. In sensitivity analyses, associations between weekly rate of GWG and birth outcomes were similar to associations with GWG z‐score (Table S2). Associations between HIV status, post‐conception ART regimen and birth outcomes did not reach significance (Table S3).

**Table 2:** Log‐binomial and linear regression models for the association between GWG z‐score and birth outcomes

			GWG z‐score (reference—medium tertile)			
	Continuous		Lower tertile		Higher tertile	
	Mean (SD)	Mean difference (95% CI)	Mean (SD)	Mean difference (95% CI)	Mean (SD)	Mean difference (95% CI)
Continuous birth weight (g)	3231 (509)	**68.53 (8.96, 128.10**)	3105 (506)	−36.57 (−182.95, 109.81)	3392 (479)	**158.15 (20.24, 296.07)**
	*N* (%)	RR (95% CI)	*N* (%)	RR (95% CI)	*N* (%)	RR (95% CI)
Low birth weight (<2500 g)	22 (8%)	0.74 (0.49, 1.13)	10 (11%)	1.28 (0.54, 3.08)	4 (4%)	0.74 (0.23, 2.39)
High birth weight (>4000 g)	12 (4%)	**2.18 (1.18, 4.01)**	4(4%)	0.40 (0.04, 3.60)	7 (7%)	1.46 (0.42, 5.03)
Small size for GA (<10th percentile)	21 (7%)	0.87 (0.57, 1.34)	7 (8%)	0.80 (0.30, 2.11)	5 (5%)	0.62 (0.21, 1.82)
Large size for GA (>90th percentile)	45 (16%)	1.15 (0.85, 1.55)	10 (11%)	0.90 (0.42, 1.96)	20 (21%)	1.22 (0.65, 2.29)
Preterm delivery (<37 weeks GA)	28 (10%)	1.03 (0.74, 1.44)	11 (13%)	1.30 (0.57, 2.98)	8 (8%)	1.12 (0.45, 2.79)

*Note*: Adjusted for age, SES, pre‐pregnancy BMI and HIV status.

Abbreviations: BMI, body mass index; GA, gestational age; SES, socio‐economic status.

### 4.3 Exploratory differences in GWG z‐score tertiles and adverse birth outcomes by HIV and ART

**Figure 3:**
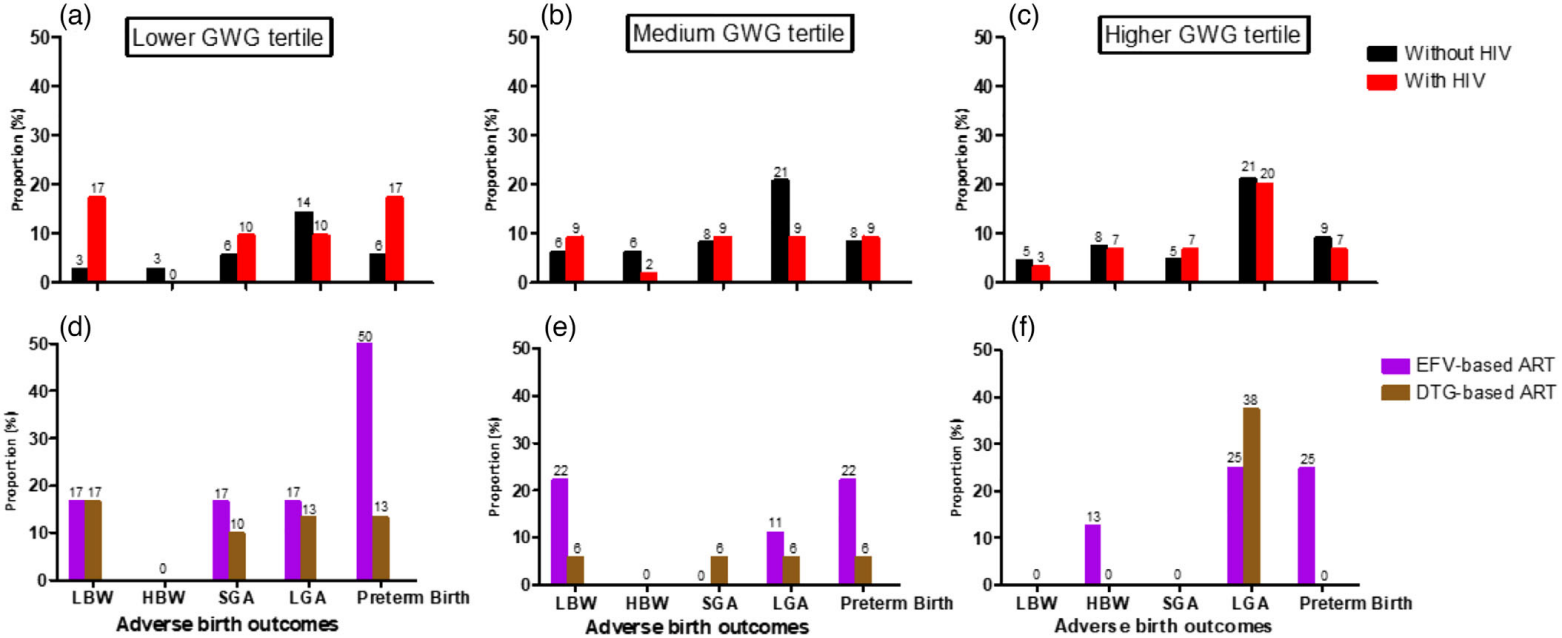
Overall proportions of adverse birth outcomes in the different GWG z‐score tertiles (lower, medium and higher), by HIV status (A–C) and post‐conception ART (D–F). HBW, high birth weight; LBW, low birth weight; LGA, large for gestational age; SGA, small for gestational age. Adverse birth outcome sample sizes for HIV status and pre‐pregnancy body mass index (BMI) comparisons are as follows: lower GWG tertile (LBW *n* = 10, HBW *n* = 1, SGA *n* = 7, LGA *n* = 10, preterm birth *n* = 11), medium GWG tertile (LBW *n* = 8, HBW *n* = 4, SGA *n* = 9, LGA *n* = 15, preterm birth *n* = 9) and higher GWG tertile (LBW *n* = 4, HBW *n* = 7, SGA *n* = 5, LGA *n* = 20, preterm birth *n* = 8). Adverse birth outcome sample sizes for pre‐conception ART comparison are as follows: lower GWG tertile (LBW *n* = 6, HBW *n* = 0, SGA *n* = 4, LGA *n* = 5, preterm birth *n* = 7), medium GWG tertile (LBW *n* = 3, HBW *n* = 0, SGA *n* = 1, LGA *n* = 2, preterm birth *n* = 3), higher GWG tertile (LBW *n* = 0, HBW *n* = 1, SGA *n* = 0, LGA *n* = 4, preterm birth *n* = 1).

We explored if the proportion of adverse birth outcomes within GWG z‐score tertiles differed by HIV status and ART regimen (Figure 3). In the lower GWG z‐score tertile, WHIV had a higher proportion of LBW (WHIV 17 vs. without HIV 3%) and PTD (WHIV 17 vs. without HIV 6%) compared to women without HIV; those initiating DTG‐based ART had a lower proportion of PTD (DTG‐based ART 13 vs. EFV‐based ART 50%) compared to women initiating EFV‐based ART. Within the higher GWG z‐score tertile, women initiating DTG‐based ART had higher LGA (DTG‐based ART 38 vs. EFV‐based ART 25%) compared to those initiating EFV‐based ART, but there were no apparent differences in adverse birth outcomes by HIV status. In sensitivity analysis, similar results were obtained with NAM categories for GWG rate (Figure S4).

## 5 DISCUSSION

In this cohort of pregnant women living with and without HIV in a peri‐urban setting in South Africa, one‐third had GWG below and almost half had GWG above NAM recommendations. GWG was positively associated with increased infant birth weight. WHIV gained weight more slowly than women without HIV; however, there was no evidence of differences in the rate of GWG observed by the ART regimen.

Increasing evidence suggest that DTG is associated with increased weight gain [3, 5, 6, 33], but there are limited data in pregnant women. In this study, we found that although WHIV gained weight more slowly than those without HIV, there were no differences in GWG between those who initiated DTG‐ versus EFV‐based ART in pregnancy. Our findings are similar to the SMARTT study in the United States which found no differences in GWG between different ART classes in the overall sample [34]. However, the stratification of participants by pre‐pregnancy BMI in the SMARTT study revealed greater GWG among women with overweight and obesity‐initiating DTG compared to other ART classes. In addition, our results are in contrast to the Tsepamo study conducted in Botswana which observed higher GWG among women who initiated DTG‐based ART than those who initiated EFV [8]. These differences might be due to a limited sample size in our study.

GWG outside NAM recommendations has been linked to adverse health outcomes in pregnancy and postpartum periods for women [11, 15, 16, 17, 18]. Overall, more than 80% of this cohort did not meet NAM's GWG standards. In particular, one‐third of our cohort had GWG below NAM recommendations, the majority of which were WHIV. Inadequate weight gain in PHIV during pregnancy is partly attributed to poor nutritional status, and dietary supplements have been shown to improve health outcomes [35, 36]. On the other hand, almost half of our cohort had GWG above the NAM recommended range, including one‐third of WHIV. These results are concerning considering that PHIV already have a higher risk of adverse birth outcomes [37, 38] and metabolic health outcomes associated with HIV‐induced chronic inflammation [39–41]. In addition, unwanted GWG in PHIV could exacerbate their poor mental health and negatively affect ART adherence [42]. Therefore, GWG monitoring efforts in this population should be encouraged, starting with the inclusion of GWG recommendations in the South African guidelines for maternity care.

The establishment of accurate associations between GWG and GA‐dependent birth outcomes requires a GWG assessment method that is not correlated with GA. We used the GWG‐for‐age z‐score, which is independent of gestation duration and accounts for non‐linearity in weight gain [22, 23]. We found that a higher GWG z‐score was positively associated with increased infant birth weight and results were similar when associations were evaluated using weekly rate of GWG. Our findings are supported by a recent meta‐analysis from 24 low‐ and middle‐income countries which showed positive associations between GWG and birth weight [43]. High levels of GWG outside NAM standards may compound childhood obesity and related adverse cardiometabolic health over the lifecourse in this setting, especially considering that the majority of the women enter pregnancy with overweight/obesity which has also been shown to be associated with infant weight in both WHIV and without HIV [44, 45, 46].

Our results should be interpreted in light of several strengths and limitations. Strengths include the availability of rich data on multiple exposures and outcomes (HIV, ART, GWG and adverse birth outcomes). In addition, this study is among the few that assessed GWG and adverse birth outcomes using both the weekly rate of GWG and z‐scores; thereby minimizing bias on GA‐related outcomes [22, 23]. Limitations include the use of self‐reported pre‐pregnancy weight for BMI calculation which might have underestimated the burden of obesity. We also had a small sample size for post‐conception DTG versus EFV comparisons; however, findings related to the rate of GWG were unchanged when assessed among both pre‐ and post‐conception ART users. Nevertheless, a sample size of 401 within each group would be needed to reach sufficient power to evaluate a clinically meaningful change in GWG related to DTG‐ versus EFV‐based ART exposure. The GWG standards used were developed from women without HIV [23, 24], which might have biased our GWG estimates for WHIV; however, no GWG exist for WHIV. Only 73% of the cohort attended the 33–38 weeks gestation visit (due to COVID‐19‐related restrictions) and hence had a GWG measure. However, there were no important differences in socio‐demographic characteristics between the full cohort and those with a 33–38 weeks gestation visit (Table S1), suggesting that possible selection bias is unlikely to meaningfully change our results.

## 6 CONCLUSIONS

In this South African cohort, we observed a large proportion of GWG below and above NAM recommendations. GWG was positively associated with infant birth weight. Although WHIV gained weight more slowly than women without HIV in the second and third trimester of pregnancy, there were no differences in the rate of GWG by ART regimen among post‐conception DTG‐ and EFV‐based ART users. Given the high levels of GWG outside of the NAM recommendations, our findings highlight the need for public health interventions aimed at monitoring GWG in women living with and without HIV to improve health outcomes.

## COMPETING INTERESTS

The authors declare that they have no competing interests.

## AUTHORS’ CONTRIBUTIONS

AMB, LM, JJ, MM, EFW, GP, SC‐U and STM conceptualized and designed the study. HPM, AF and DM collected the data. HPM, HG and DM performed the analyses. HPM interpreted the data, searched the literature and wrote the manuscript. All authors reviewed, edited and approved the final manuscript.

## FUNDING

This work was supported by the Providence/Boston Center for AIDS Research (P30AI042853), the Population Studies and Training Center at Brown University (P2CHD041020), the National Institute of Diabetes and Digestive and Kidney Diseases (NIDDK U01‐DK‐18‐018/019) and the Fogarty International Center at the National Institutes of Health (R21TW011678).

## PATIENT CONSENT STATEMENT

All women provided written informed consent prior to study participation.

## Supporting information


**Table S1**. Characteristics of women included in the analysis, overall and stratified by maternal HIV status
**Table S2**. Log‐binomial and linear regression models for the association between weekly rate of GWG and birth outcomes
**Table S3**. Log‐binomial and linear regression models for the association between HIV status (n = 377), post‐conception ART regimen (n = 105) and birth outcomes
**Figure S1**. Overall distribution of weekly rate of GWG overall (A: n = 292) and by HIV status (B: 153 without HIV, C: 139 WHIV) and post‐conception ART (D: 20 EFV‐based ART, E: 56 DTG‐based ART). The dotted lines show a reference range for GWG rate ‘within’ NAM standards across all BMI categories. ART, antiretroviral therapy, EFV, efavirenz, DTG dolutegravir.
**Figure S2**. Distribution of GWG z‐score between 24–28 weeks gestation and 33–38 weeks gestation by HIV status (A: 153 without HIV, B: 139 WHIV) and post‐conception ART (C: 20 EFV‐based ART, D: 56 DTG‐based ART). ART, antiretroviral therapy; EFV, efavirenz; DTG, dolutegravir.
**Figure S3**. Relationship between GWG z‐score and weekly rate of GWG When the z‐score = −1 (16th percentile) then weekly rate of GWG = 0.28kg/week (95% CI = 0.23, 0.32), when z‐score = 0 (50th percentile), then weekly rate of GWG = 0.43 kg/week (95% CI = 0.39, 0.47), when the z‐score = 1 (84th percentile) then weekly rate of GWG = 0.58kg/week (95% CI = 0.51, 0.65).
**Figure S4**. Overall proportions of adverse birth outcomes in the different weekly rate of GWG NAM categories (below, within and above), by HIV status (A‐C) and post‐conception ART (D‐F). LBW (low birth weight), HBW (high birth weight), SGA (small for gestational age), LGA (large for gestational age). Adverse birth outcome sample sizes for HIV status comparison are as follows: slower weekly rate of GWG (LBW n = 10, HBW n = 3, SGA n = 11, LGA n = 14, preterm birth n = 9), normal weekly rate of GWG (LBW n = 2, HBW n = 0, SGA n = 4, LGA n = 7, preterm birth n = 5) and faster weekly rate of GWG (LBW n = 10, HBW n = 9, SGA n = 6, LGA n = 24, preterm birth n = 14). Adverse birth outcome sample sizes for pre‐conception ART comparison are as follows: slower weekly rate of GWG (LBW n = 4, HBW n = 0, SGA n = 2, LGA n = 5, preterm birth n = 5), normal weekly rate of GWG (LBW n = 1, HBW n = 0, SGA n = 1, LGA n = 1, preterm birth n = 1), faster weekly rate of GWG (LBW n = 4, HBW n = 1, SGA n = 2, LGA n = 5, preterm birth n = 5).

## Data Availability

All data generated or analysed during this study are included in this published article (and its supplementary information files). In addition, the datasets used and/or analysed during the current study are available from the corresponding author on reasonable request.
