# Supplementary material for: Gestational weight gain and adverse birth outcomes in South African women with HIV on antiretroviral therapy and without HIV: a prospective cohort study
Source: J Int AIDS Soc. 2024 Jun 26;27(6):e26313. doi: 10.1002/jia2.26313 (PMC11208166; doi:10.1002/jia2.26313)
Supplement: Supplementary file 1 — Table S1. Characteristics of women included in the analysis, overall and stratified by maternal HIV status Table S2. Log‐binomial and linear regression models for the association between weekly rate of GWG and birth outcomes Table S3. Log‐binomial and linear regression models for the association between HIV status (n = 377), post‐conception ART regimen (n = 105) and birth outcomes Figure S1. Overall distribution of weekly rate of GWG overall (A: n = 292) and by HIV status (B: 153 without HIV, C: 139 WHIV) and post‐conception ART (D: 20 EFV‐based ART, E: 56 DTG‐based ART). The dotted lines show a reference range for GWG rate ‘within’ NAM standards across all BMI categories. ART, antiretroviral therapy, EFV, efavirenz, DTG dolutegravir. Figure S2. Distribution of GWG z‐score between 24–28 weeks gestation and 33–38 weeks gestation by HIV status (A: 153 without HIV, B: 139 WHIV) and post‐conception ART (C: 20 EFV‐based ART, D: 56 DTG‐based ART). ART, antiretroviral therapy; EFV, efavirenz; DTG, dolutegravir. Figure S3. Relationship between GWG z‐score and weekly rate of GWG When the z‐score = −1 (16th percentile) then weekly rate of GWG = 0.28kg/week (95% CI = 0.23, 0.32), when z‐score = 0 (50th percentile), then weekly rate of GWG = 0.43 kg/week (95% CI = 0.39, 0.47), when the z‐score = 1 (84th percentile) then weekly rate of GWG = 0.58kg/week (95% CI = 0.51, 0.65). Figure S4. Overall proportions of adverse birth outcomes in the different weekly rate of GWG NAM categories (below, within and above), by HIV status (A‐C) and post‐conception ART (D‐F). LBW (low birth weight), HBW (high birth weight), SGA (small for gestational age), LGA (large for gestational age). Adverse birth outcome sample sizes for HIV status comparison are as follows: slower weekly rate of GWG (LBW n = 10, HBW n = 3, SGA n = 11, LGA n = 14, preterm birth n = 9), normal weekly rate of GWG (LBW n = 2, HBW n = 0, SGA n = 4, LGA n = 7, preterm birth n = 5) and faster weekly rate of GWG (LBW n = 10, HBW n [file JIA2-27-e26313-s001.docx]

**SUPPLEMENTARY MATERIAL**

**Table S1.** Characteristics of women included in the analysis, overall and stratified by maternal HIV status

|  |  |  | HIV status | | p-value |
| --- | --- | --- | --- | --- | --- |
| **Characteristic** | Subset with GWG  N = 292 | Overall cohort  N = 400 | Without HIV  N = 200 | With HIV  N = 200 |  |
| Age (years)  Median (IQR) | 29 (25-33) | 30 (25-34) | 27 (24-31) | 31 (27-36) | <0.01 |
| Gestational age at first ANC (weeks)  Median (IQR) | 17 (12-21) | 17 (13-21) | 17 (13-22) | 17 (12-21) | 0.20 |
| Gestational age at study enrolment (weeks)  Median (IQR) | 26 (24-27) | 26 (24-27) | 26 (24-27) | 26 (24-27) | 0.83 |
| Weight (kg), median (IQR)  Pre-pregnancy  24-28 weeks GA  33-38 weeks GA | 80 (65-93)  84 (71-98)  87 (75-100) | 78 (65-90)  82 (69-96)  87 (75-100) | 80 (65-90)  86 (73-97)  89 (78-101) | 76 (63-91)  80 (68-95)  83 (71-100) | 0.10  0.04  0.04 |
| Pre-pregnancy BMI (kg/m^2^)  Underweight (<18.5)  Normal (18.5-24.9)  Overweight (25-29.9)  Obese (≥30)  Median (IQR) | 0 (0)  45 (15)  82 (28)  165 (57)  31 (26-36) | 0 (0)  79 (20)  113 (28)  207 (52)  30 (26-35) | 0 (0)  37 (18)  51 (25)  111 (56)  31 (26-36) | 0 (0)  42 (21)  62 (31)  96 (48)  29 (25-34) | 0.32 |
| Parity  Primiparous  Multiparous  Median (IQR) | 58 (20)  234 (80)  2 (2-3) | 74 (18)  326 (82)  3 (2-3) | 51 (25)  149 (75)  2 (1-3) | 23 (12)  177 (88)  3 (2-4) | 0.01 |
| Education  Primary  Partially completed high school  Completed high school  Tertiary | 5 (1)  177 (61)  93 (32)  17 (6) | 10 (3)  236 (59)  133 (33)  21 (5) | 3 (1)  102 (51)  81 (41)  14 (7) | 7 (3)  134 (67)  52 (26)  7 (4) | 0.01 |
| Socio-economic status  Lower  Middle  Higher | 96 (33)  76 (26)  120 (41) | 129 (32)  109 (27)  162 (41) | 51 (25)  58 (29)  91 (46) | 78 (38)  51 (26)  71 (36) | 0.01 |
| Relationship status  Not in relationship  Not married/cohabiting  Married/cohabiting | 24 (8)  143 (49)  125 (43) | 32 (8)  197 (49)  171 (43) | 12 (5)  103 (52)  85 (43) | 20 (10)  94 (47)  86 (43) | 0.30 |
| Perceived food insecurity  No  Yes | 241 (83)  51 (17) | 333 (83)  67 (17) | 172 (86)  28 (14) | 161 (80)  39 (20) | 0.14 |
| Hazardous alcohol use  No  Yes | 274 (94)  18 (6) | 376 (94)  24 (6) | 188 (94)  12 (6) | 188 (94)  12 (6) | 0.52 |
| Gestational weight gain (kg/week)  Below NAM standards  Within NAM standards  Above NAM standards  Median (IQR) | 101 (35)  50 (17)  141 (48)  0.30 (0.12-0.50) | 101 (35)  50 (17)  141 (48)  0.30 (0.12-0.50) | 41 (27)  22 (14)  90 (59)  0.37 (0.22-0.63) | 60 (43)  28 (20)  51 (37)  0.25 (0.08-0.42) | <0.01 |
| **Maternal HIV characteristics** | | | | | |
|  | Subset with GWG  N = 139 | Total participants living with HIV (n=200) | EFV-based ART (n=107) | DTG-based ART (n=86) | p-value |
| ART initiation timing  Pre-conception  Post-conception | 63 (45) 76 (55) | 88 (44)  112 (56) | 71 (66)  36 (34) | 11 (13)  75 (87) | <0.01 |
| Duration on ART (months)  Median (IQR) | 3 (1-50) | 3 (1-51) | 43 (3-77) | 1 (1-2) | <0.01 |
| CD4 count (cells/µL)  ≤350  351-500  >500  Median (IQR) | 27 (19)  30 (22)  52 (37)  463 (352-692) | 47 (23)  42 (21)  70 (35)  461 (324-621) | 22 (21)  30 (28)  47 (44)  491 (356-670) | 23 (27)  12 (14)  21 (24)  410 (260-585) | <0.01 |
| Viral Load (copies/mL)  Undetectable (<50)  Detectable (≥50)  Median (IQR) | 45 (32)  19 (14)  15 (15-97) | 62 (31)  27 (14)  15 (15-89) | 58 (54)  18 (17)  15 (15-50) | 1 (1)  7 (8)  2104 (131-134789) | <0.01 |
| Weight (kg), median (IQR)  Pre-pregnancy  24-28 weeks GA  33-38 weeks GA | 78 (65-95)  81 (69-98)  83 (71-100) | 76 (73-91)  80 (68-94)  83 (71-100) | 75 (62-91)  81 (69-95)  86 (72-100) | 76 (65-90)  77 (65-94)  82 (70-98) | 0.95  0.28  0.38 |
| Gestational weight gain (kg/week)  Below NAM standards  Within NAM standards  Above NAM standards  Median (IQR) | 60 (43)  28 (20)  51 (37)  0.25 (0.08-0.43) | 60 (43)  28 (20)  51 (37)  0.25 (0.08-0.43) | 34 (46)  16 (21)  25 (33)  0.26 (0.03-0.42) | 26 (41)  12 (18)  26 (41)  0.25 (0.11-0.42) | 0.67 |
| Missing data: gestational age at first ANC n=8, pre-pregnancy weight n=1, 24-28 weeks GA weight n=1, 33-38 weeks GA weight n=98, pre-pregnancy BMI n=1, GWG n=108, CD4 count n=41, viral load n=111. For maternal HIV characteristics - 7 participants were on PI-based ART, missing GWG n=61. ANC - antenatal care, GA - gestational age, BMI - body mass index, NAM - National Academy of Medicine, ART - antiretroviral therapy, EFV - efavirenz, DTG - dolutegravir | | | | | |

**Figure S1.** Overall distribution of weekly rate of GWG overall (**A**: n=292) and by HIV status (**B**: 153 without HIV, **C**: 139 WHIV) and post-conception ART (**D**: 20 EFV-based ART, **E**: 56 DTG-based ART). The dotted lines show a reference range for GWG rate ‘within’ NAM standards across all BMI categories. ART - antiretroviral therapy, EFV - efavirenz, DTG - dolutegravir

**Figure S2.** Distribution of GWG z-score between 24-28 weeks gestation and 33-38 weeks gestation by HIV status (**A**: 153 without HIV, **B**: 139 WHIV) and post-conception ART (**C**: 20 EFV-based ART, **D**: 56 DTG-based ART). ART - antiretroviral therapy, EFV - efavirenz, DTG - dolutegravir


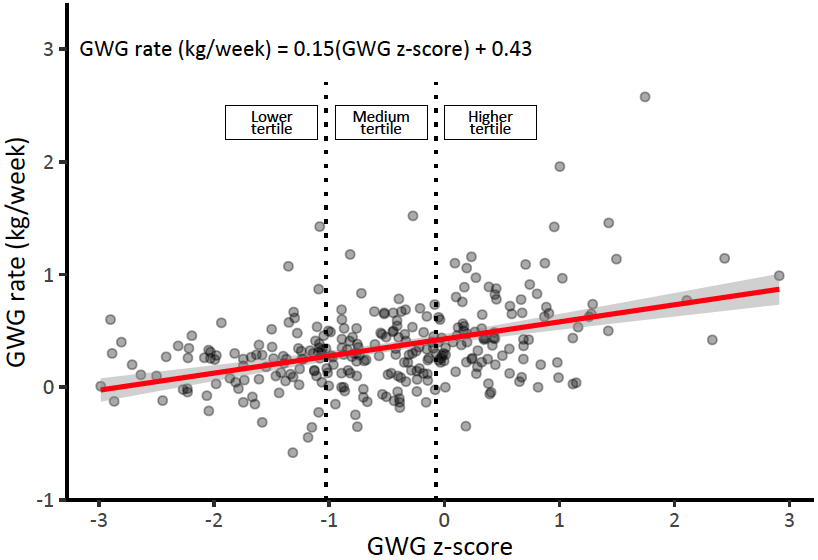


**Figure S3.** Relationship between GWG z-score and weekly rate of GWG

When the z-score = -1 (16^th^ percentile) then weekly rate of GWG = 0.28kg/week (95% CI = 0.23, 0.32), when z-score = 0 (50^th^ percentile), then weekly rate of GWG = 0.43 kg/week (95% CI = 0.39, 0.47), when the z-score = 1 (84^th^ percentile) then weekly rate of GWG = 0.58kg/week (95% CI = 0.51, 0.65)

| **Table S2.** Log-binomial and linear regression models for the association between weekly rate of GWG and birth outcomes | | | | | | |
| --- | --- | --- | --- | --- | --- | --- |
|  |  | | GWG rate (reference – within NAM standards) | | | |
|  | Continuous | | Below NAM standards | | Above NAM standards | |
|  | Mean (SD) | Mean difference  (95% CI) | Mean (SD) | Mean difference  (95% CI) | Mean (SD) | Mean difference  (95% CI) |
| Continuous birth weight (g) | 3231 (509) | **172.80 (9.55, 336.05)** | 3139 (544) | -82.85 (-235.82, 70.12) | 3308 (502) | 48.33 (-95.03, 191.68) |
|  | N (%) | RR (95% CI) | N (%) | RR (95% CI) | N (%) | RR (95% CI) |
| Low birth weight (<2500 g) | 22 (8%) | 1.18 (0.38, 3.63) | 10 (10%) | 2.77 (0.64, 11.91) | 10 (7%) | 3.05 (0.70, 13.67) |
| High birth weight (>4000 g) | 12 (4%) | 2.15 (0.74, 6.23) | 3 (3%) | not estimable | 9 (7%) | not estimable |
| Small size for GA (<10^th^ percentile) | 21 (7%) | 0.52 (0.13, 2.03) | 11 (11%) | 1.36 (0.46, 4.04) | 6 (4%) | 0.53 (0.15, 1.87) |
| Large size for GA (>90^th^ percentile) | 45 (16%) | 1.24 (0.59, 2.58) | 14 (14%) | 1.01 (0.44, 2.30) | 24 (18%) | 1.15 (0.53, 2.49) |
| Preterm delivery (<37 weeks GA) | 28 (10%) | 1.62 (0.74, 3.57) | 9 (9%) | 0.91 (0.33, 2.26) | 14 (10%) | 1.39 (0.52, 3.71) |
| Adjusted for age, SES, pre-pregnancy BMI and HIV status. NAM - National Academy of Medicine, GA - gestational age, SES - socioeconomic status, BMI - body mass index | | | | | | |

| **Table S3.** Log-binomial and linear regression models for the association between HIV status (n=377), post-conception ART regimen (n=105) and birth outcomes | | | | |
| --- | --- | --- | --- | --- |
|  | ^†^HIV status  (reference – without HIV) | | ^‡^Post-conception ART  (reference – EFV-based) | |
|  | With HIV | | DTG-based ART | |
|  | Mean (SD) | Mean difference  (95% CI) | Mean (SD) | Mean difference  (95% CI) |
| Continuous birth weight (g) | 3049 (611) | -56.65 (-182.59, 69.29) | 3065 (668) | 83.21 (-199.98, 366.40) |
|  | N (%) | RR (95% CI) | N (%) | RR (95% CI) |
| Low birth weight (<2500 g) | 32 (17%) | 1.16 (0.64, 2.12) | 16 (22%) | 1.10 (0.53, 2.26) |
| High birth weight (>4000 g) | 4 (2%) | 0.40 (0.12, 1.32) | 1 (1%) | 0.65 (0.04, 9.90) |
| Small size for GA (<10^th^ percentile) | 15 (8%) | 1.13 (0.53, 2.39) | 5 (7%) | 0.42 (0.06, 3.11) |
| Large size for GA (>90^th^ percentile) | 20 (11%) | 0.65 (0.38, 1.14) | 8 (11%) | 0.84 (0.26, 2.71) |
| Preterm delivery (<37 weeks GA) | 35 (19%) | 0.80 (0.48, 1.32) | 14 (19%) | 0.74 (0.38, 1.45) |
| ^†^ Adjusted for age, SES and pre-pregnancy BMI; ^‡^ Adjusted for age and CD4 count**.**  NAM - National Academy of Medicine, GA - gestational age, SES - socioeconomic status, BMI - body mass index | | | | |


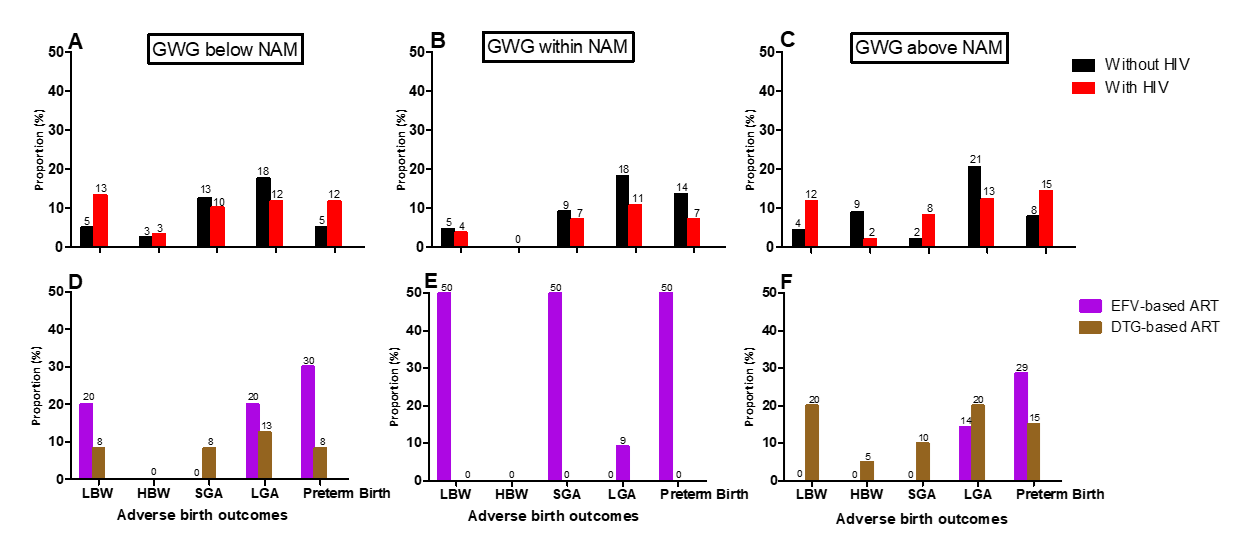


**Figure S4.** Overall proportions of adverse birth outcomes in the different weekly rate of GWG NAM categories (below, within and above), by HIV status (A-C) and post-conception ART (D-F). LBW (low birth weight), HBW (high birth weight), SGA (small for gestational age), LGA (large for gestational age). Adverse birth outcome sample sizes for HIV status comparison are as follows: slower weekly rate of GWG (LBW n=10, HBW n=3, SGA n=11, LGA n=14, preterm birth n=9), normal weekly rate of GWG (LBW n=2, HBW n=0, SGA n=4, LGA n=7, preterm birth n=5) and faster weekly rate of GWG (LBW n=10, HBW n=9, SGA n=6, LGA n=24, preterm birth n=14). Adverse birth outcome sample sizes for pre-conception ART comparison are as follows: slower weekly rate of GWG (LBW n=4, HBW n=0, SGA n=2, LGA n=5, preterm birth n=5), normal weekly rate of GWG (LBW n=1, HBW n=0, SGA n=1, LGA n=1, preterm birth n=1), faster weekly rate of GWG (LBW n=4, HBW n=1, SGA n=2, LGA n=5, preterm birth n=5)
